# Hepatitis E Virus Infection in HIV-infected Persons

**DOI:** 10.3201/eid1803.111278

**Published:** 2012-03

**Authors:** Nancy F. Crum-Cianflone, Jennifer Curry, Jan Drobeniuc, Amy Weintrob, Michael Landrum, Anuradha Ganesan, William Bradley, Brian K. Agan, Saleem Kamili, The Infectious Disease Clinical Research Program HIV Working Group

**Affiliations:** Uniformed Services University of the Health Sciences, Bethesda, Maryland, USA (N.F. Crum-Cianflone, J. Curry, A. Weintrob, M. Landrum, A. Ganesan, W. Bradley, B.K. Agan);; Naval Medical Center San Diego, San Diego, California, USA (N.F. Crum-Cianflone);; Naval Medical Center Portsmouth, Portsmouth, Virginia, USA (J. Curry);; Centers for Disease Control and Prevention, Atlanta, Georgia, USA (J. Drobeniuc S. Kamili);; Walter Reed Army Medical Center, Washington DC, USA (A. Weintrob);; San Antonio Military Medical Center, San Antonio, Texas, USA (M. Landrum);; National Naval Medical Center, Bethesda (A. Ganesan)

**Keywords:** Hepatitis E virus, HIV, epidemiology, hepatitis, military, viruses

## Abstract

To determine whether hepatitis E virus (HEV) is a cause of hepatitis among HIV-infected persons, we evaluated 1985–2009 data for US military beneficiaries. Evidence of acute or prior HEV infection was detected for 7 (4%) and 5 (3%) of 194 HIV-infected persons, respectively. HEV might be a cause of acute hepatitis among HIV-infected persons.

Among immunosuppressed persons in industrialized countries, hepatitis E virus (HEV) is a cause of sporadic acute viral hepatitis and chronic hepatitis (*1**,**2*). In the United States, liver test results are often abnormal for HIV-infected persons; however, few studies have evaluated whether HEV is a cause of hepatitis in this population (*3*).

## The Study

**Figure 1:**
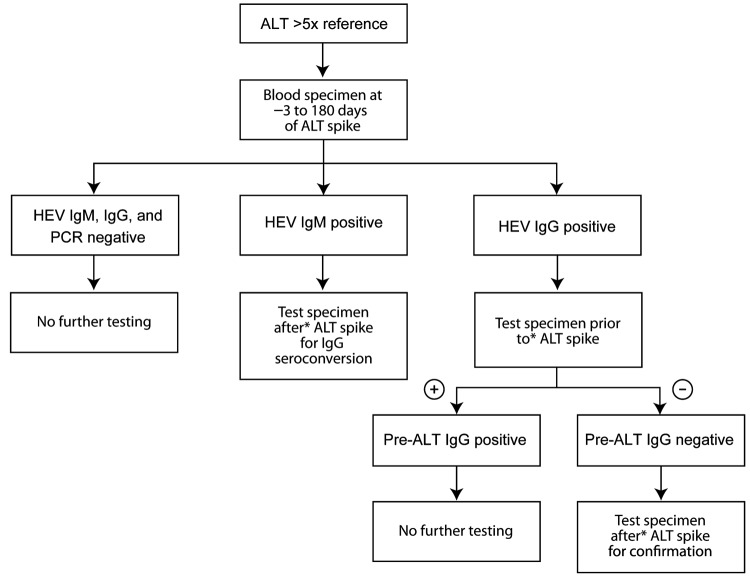
Testing strategy for acute hepatitis E virus (HEV) infection among US military beneficiaries who had had increased alanine aminotransferase (ALT) levels during 1985–2009. +, positive; –, negative.

We retrospectively evaluated HIV-infected persons for whom alanine aminotransferase (ALT) levels had increased acutely (>5 × the upper limit of normal) during the HIV epidemic (1985–2009). Eligible participants were US military beneficiaries (persons entitled to receive care at a military treatment facility) for whom a stored serum specimen, collected from 3 days before ALT increase through 180 days after ALT increase, was available for HEV testing. A case of acute HEV infection was defined as a sample with HEV RNA and/or IgM against HEV or evidence of IgG seroconversion. All samples collected at the time of ALT increase were tested for IgM and IgG against HEV by using commercially available enzyme immunoassays (Diagnostics Systems, Nizhniy Novgorod, Russia) (*4*) and PCR for HEV RNA (*5*). The testing strategy is shown in Figure 1. All positive results were verified by retesting.

Statistical analyses included descriptive statistics presented as numbers (percentages) for categorical variables and medians (interquartile ranges [IQRs]) for continuous variables. The percentage of participants with HEV infection was defined as the number with an initial positive result for IgM or IgG against HEV divided by the total number of evaluable study participants. To compare characteristics among those with and without HEV infection, we used Fisher exact testing for categorical variables and rank-sum testing for continuous variables. A multivariate logistic regression model was used to identify factors associated with HEV infection. All analyses were performed by using Stata version 10.0 (StataCorp LP, College Station, TX, USA).

Among 4,410 HIV-infected persons, 458 (10%) had increased ALT levels at least 1 time during 32,468 person-years of follow-up. Among these, serum samples were available for HEV testing for 194 (42%) participants, among whom median age was 34 (IQR 30–40) years, 95% were male, and 42% were white (Table 1). The median ALT level was 440 (IQR 322–812) IU/mL. At the ALT spike, participants had been infected with HIV for a median duration of 5 (IQR 2–9) years; median CD4 cell count was 436 (IQR 239–627) cells/mm^3^, median plasma HIV RNA level was 13,581 (IQR 762–71,586) copies/mL, and 28% of participants were receiving antiretroviral therapy.

**Table 1:** Characteristics of 194 HIV-positive US military beneficiaries at time of ALT increase, 1985–2009*

Characteristic†	Total cohort	HEV seropositive,‡ n = 13	HEV seronegative, n = 181	Odds ratio	p value
Demographics					
Age, y	34 (30–40)	35 (32–40)	34 (29–40)	1.01	0.66
Male gender	185 (95)	13 (100)	172 (95)	–	–
Ethnicity					0.4
White	82 (42)	7 (54)	75 (41)	Referent	
African American	77 (40)	4 (31)	73 (40)	0.59	
Hispanic	29 (15)	1 (8)	28 (16)	0.38	
Other	6 (3)	1 (8)	5 (3)	2.14	
Military status					0.32
Active duty	98 (50)	4 (31)	94 (52)	Referent	
Retired	85 (44)	8 (61)	77 (43)	2.44	
Spouse/dependent	11 (6)	1 (8)	10 (5)	2.35	
Overseas travel§	48/127 (38)	1/5 (20)	47/122 (39)	0.4	0.65
Liver function test results					
Timing of blood collection after ALT increase, d	27 (0–104)	31 (7–107)	23 (0–105)	1.0	0.78
ALT level, IU/L	440 (322–812)	367 (241–483)	454 (333–821)	0.99	0.63
AST level, IU/L	262 (183–653)	297 (152–474)	260 (185–693)	1.0	0.66
Clinical conditions					
Gonorrhea§	54 (28%	2 (15)	52 (29)	0.44	0.36
Chlamydia/nonspecific urethritis§	20 (10)	1 (8)	19 (11)	0.7	1.0
Syphilis§	32 (17)	4 (31)	28 (16)	2.38	0.24
Any STI§¶	84 (44)	6 (46)	78 (44)	1.1	1.0
Hepatitis B#					
Prior infection	97 (51)	8 (62)	89 (50)	1.6	0.57
Chronic	30 (15)	3 (23)	27 (15)	1.69	0.43
Hepatitis C#	12 (6)	2 (15)	10 (6)	3.05	0.19
HIV–specific factors					
HIV infection duration, y	5 (1.8–8.8)	5.3 (2.3–10.0)	4.9 (1.7–8.6)	1.01	0.89
CD4 cell count, cells/mm^3^	436 (239–627)	217 (9–589)	439 (258–633)	0.79	0.07
<200	40 (21)	6 (46)	34 (19)	Referent	–
200–499	80 (41)	3 (23)	77 (42)	0.22	0.06
>500	74 (38)	4 (31)	70 (39)	0.32	0.10
Median HIV RNA level, log_10_ copies/mL§	4.1 (2.9–4.9)	4.7 (3.9–5.4)	4.1 (2.9–4.8)	1.96	0.04
HIV RNA copies/mL					
<1,000	48 (27)	1 (9)	47 (28)	Referent	–
1,000–10,000	36 (20)	2 (18)	34 (20)	2.76	0.57
>10,000	96 (53)	8 (73)	88 (52)	4.27	0.27
Antiretroviral drug use	55 (28)	1 (8)	54 (30)	0.2	0.12

*ALT, alanine aminotransferase; HEV, hepatitis E virus; AST, aspartate aminotransferase; STI, sexually transmitted infection. †Characteristics are expressed as number (percent) for categorical variables and medians (interquartile range) for continuous variables. ‡IgM and/or IgG against HEV. §Some data were missing: for overseas travel n = 127; STIs n = 191; HIV RNA level n = 180. ¶Gonorrhea, chlamydial infection, nonspecific urethritis, or syphilis. #Based on clinical diagnoses; similar results noted when prior hepatitis B virus infection was defined as total positive for hepatitis B virus core antigen, chronic hepatitis B infection as positive for hepatitis B virus surface antigen, and hepatitis C infection as positive for IgG against hepatitis C virus.

Samples for HEV testing were available at a median of 27 (IQR 0–104) days after the increase in ALT level. For 13 (6.7%) participants, IgM and/or IgG against HEV were present at the time closest to the ALT increase; antibody prevalence among those with elevated ALT levels did not increase during the HIV epidemic (χ^2^ 0.76, p = 0.68). The 13 HIV-infected persons who were HEV seropositive (IgM or IgG at ALT spike) were similar to the 181 who were HEV seronegative in terms of demographics, military duty status, laboratory data, and overseas travel (Table 1). HEV-seropositive persons had higher plasma HIV RNA levels (4.7 vs. 4.1 log_10_ copies/mL, p = 0.04) and the association with lower CD4 cell counts was borderline (median 217 vs. 439 cells/mm^3^, p = 0.07). In the multivariate logistic regression model adjusted for age, plasma HIV RNA levels remained significantly associated with HEV seropositivity (odds ratio 1.96 per log_10_, 95% CI 1.04–3.71, p = 0.04).

**Figure 2:**
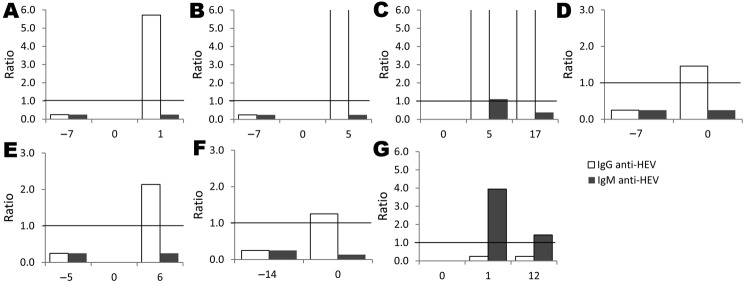
IgM and IgG against hepatitis E virus (HEV) signal/cutoff ratios for 7 HIV-infected US military beneficiaries with acute HEV infection, 1985–2009. Serum specimens were tested for HEV markers before and after alanine aminotransferase spike, indicated by 0.0 on x-axis. Horizontal lines indicate enzyme immunoassay signal/cutoff ratio of 1.0.

Additional testing was conducted for all 13 participants with IgM or IgG against HEV or with HEV RNA at the time of ALT increase (Figure 1). HEV RNA was detected in 1 participant who also seroconverted (IgG) at the time of ALT increase. According to samples collected at or near ALT spike and after ALT spike, 5 more participants seroconverted. One participant had IgM detectable in all 3 samples (at or near ALT spike, after ALT spike, and at follow-up); the participant did not seroconvert, and HEV RNA was not detectable in any sample. In total, 7 (3.6%, 95% CI 1.6%–7.6%) of the 194 HIV-infected persons had evidence of acute HEV infection at time of ALT spike (Figure 2). HEV was deemed not to be the cause of the ALT spike for 5 participants with evidence of prior HEV infection because IgG positivity preceded the ALT increase**.** For 1 participant, IgM was found in only 1 sample; all other samples were negative for IgM, IgG, and HEV RNA. Because of unconfirmed positive follow-up results, this participant was excluded from further analysis.

For the 7 participants with acute HEV infection (Table 2), HEV was not considered during the ALT increase, and HEV testing was not conducted as part of clinical care. No significant differences in clinical or laboratory characteristics were found among the 7 participants with acute HEV infection and those without evidence of HEV infection (data not shown). Chronic HEV infection did not develop in any participant.

**Table 2:** Characteristics of HIV-positive US military beneficiaries with acute HEV infection at time of ALT increase, 1985–2009*

Patient	1	2	3	4	5	6	7
Age, y	34	35	35	33	44	41	30
Ethnicity	White	African-American	White	African-American	White	African-American	African-American
Duty status	Active	Retired	Active	Retired	Retired	Retired	Retired
Year of ALT increase	2001	1995	2000	2006	1989	1996	1996
Clinical presentation	Nausea, vomiting, abdominal pain, pale stools, dark urine	Fever, malaise, anorexia, diarrhea, dark urine, icterus	Fever, nausea, vomiting, diarrhea, abdominal pain, loss of appetite, malaise	Jaundice	Abdominal pain	Asymp	Asymp
Peak ALT, U/L	489	2,540	282	2,829	229	477	226
AST, U/L	354	988	174	4,273	209	508	130
Alkaline phosphatase, U/L	80	153	99	409	157	125	137
Total bilirubin, mg/dL	3.2	5.0	1.9	5.3	1.6	0.5	1.2
Antibodies against							
Hepatitis B virus core antigen	Neg	Pos	Pos	Pos	Neg	Pos	Pos
Hepatitis B virus surface antigen	Neg	Neg	Neg	Pos	Neg	Pos	Neg
Hepatitis C virus	Neg	Neg	Neg	Neg	Neg	Neg	Pos
History of STI since HIV Infection	None	None	None	Syphilis and chlamydia infections	Gonorrhea	Syphilis	Gonorrhea
Travel overseas	NK	NK	Kuwait	NK	NK	NK	NK
Duration of HIV, y	11	2	<1	13	2	8	9
CD4 count, cells/mm^3^	822	517	660	454	753	98	217
HIV RNA level, copies/mL	427	52,929	6,854	52,682	40,000	430,946	8,068
HAART received	Yes	No	No	No	No	No†	No‡
HEV serostatus	IgG sero and HEV RNA	IgG sero	IgM and IgG positivity	IgG sero	IgG sero	IgG sero	IgM with persistent positivity

*All patients were male; none had evidence of acute hepatitis A virus infection or chronic HEV. HEV, hepatitis E infection; ALT, alanine aminotransferase; asymp, asymptomatic; AST, aspartate aminotransferase; neg, negative; pos, positive; STI, sexually transmitted infection; NK, none known; HAART, highly active antiretroviral therapy; sero, seroconversion. †Patient was receiving monotherapy with zalcitabine. ‡Patient was receiving dual therapy with stavudine and ritonavir.

## Conclusions

HEV infection accounted for 4% of acute liver abnormalities among HIV-infected persons. Overall, HEV was detected in 6% of HIV-infected participants, similar to the 5%–21% reported earlier from the United States (*1**,**6*). Because study participation was limited to persons who had a sample available for HEV testing near the time of ALT increase, we might have missed cases of HEV infection. Overall, on the basis of our study and data from other industrialized countries (*7**,**8*), HEV is a cause of liver abnormalities in HIV-infected persons but does not seem to be more common in this population than in the general population.

HEV seropositivity did not increase over the course of the HIV epidemic. Despite increasing reports of HEV among HIV-infected persons and the general population (*1**,**3**,**7**–**10*), this increase is probably associated with increased recognition and testing. Recent studies in the United States and Europe have shown that HEV seroprevalence is stable or decreasing (*1**,**11*).

HEV infections among HIV-infected persons have been reported (*3**,**7**–**9**,**12*); however, whether this population is at increased risk for HEV infection remains uncertain. Recent studies from Europe suggest that HIV-infected persons or other immunocompromised persons are not at increased risk of acquiring HEV infections (*7**–**9**,**12*). Nonetheless, these groups are at higher risk for chronic HEV infection (*2**,**7**,**13*).

We propose that a diagnosis of HEV infection be considered for persons with viral-like hepatitis. Serologic test results may be negative despite ongoing HEV infection; hence, for HIV-infected persons (especially those with low CD4 cell counts), PCR testing for HEV RNA should be conducted (*7*). Because HEV infection may be fulminant in the presence of underlying liver disease (common among HIV-infected persons) (*14*) and may lead to chronic infection in immunosuppressed persons (*2**,**13*), testing should be considered for these persons as treatment options for HEV infection evolve. Moreover, chronic HEV infection may be averted by reducing the level of immunosuppression (*15*) and use of highly active antiretroviral therapy (*3**,**8*), but more data are needed to support these measures.

HEV infection is a newly defined cause of acute liver dysfunction among HIV-infected persons in the United States. HEV infections do not seem to preferentially occur among HIV-infected persons, suggesting that HIV itself may not be a risk factor for HEV acquisition. HEV infection should be considered among HIV-infected persons with liver abnormalities of unclear etiology.
